# An empirical ethics study of the coherence of NICE technology appraisal policy and its implications for moral justification

**DOI:** 10.1186/s12910-024-01016-0

**Published:** 2024-03-06

**Authors:** Victoria Charlton, Michael DiStefano

**Affiliations:** 1 Department of Global Health and Social Medicine, King’s College London, London, UK; 2 Department of Clinical Pharmacy, University of Colorado Anschutz Medical Campus, Aurora, USA

**Keywords:** Healthcare priority-setting, Ethics, Justice, Health technology assessment

## Abstract

**Background:**

As the UK’s main healthcare priority-setter, the National Institute for Health and Care Excellence (NICE) has good reason to want to demonstrate that its decisions are morally justified. In doing so, it has tended to rely on the moral plausibility of its principle of cost-effectiveness and the assertion that it has adopted a fair procedure. But neither approach provides wholly satisfactory grounds for morally defending NICE’s decisions. In this study we adopt a complementary approach, based on the proposition that a priority-setter's claim to moral justification can be assessed, in part, based on the coherence of its approach and that the reliability of any such claim is undermined by the presence of dissonance within its moral system. This study is the first to empirically assess the coherence of NICE’s formal approach and in doing so to generate evidence-based conclusions about the extent to which this approach is morally justified.

**Methods:**

The study is grounded in the theory, methods and standards of empirical bioethics. Twenty NICE policy documents were coded to identify and classify the normative commitments contained within NICE technology appraisal policy as of 31 December 2021. Coherence was systematically assessed by attempting to bring these commitments into narrow reflective equilibrium (NRE) and by identifying sources of dissonance.

**Findings:**

Much of NICE policy rests on coherent values that provide a strong foundation for morally justified decision-making. However, NICE’s formal approach also contains several instances of dissonance which undermine coherence and prevent NRE from being fully established. Dissonance arises primarily from four sources: i) NICE’s specification of the principle of cost-effectiveness; ii) its approach to prioritising the needs of particular groups; iii) its conception of reasonableness in the context of uncertainty, and iv) its concern for innovation as an independent value.

**Conclusion:**

At the time of analysis, the level of coherence across NICE policy provides reason to question the extent to which its formal approach to technology appraisal is morally justified. Some thoughts are offered on why, given these findings, NICE has been able to maintain its legitimacy as a healthcare priority-setter and on what could be done to enhance coherence.

**Supplementary Information:**

The online version contains supplementary material available at 10.1186/s12910-024-01016-0.

## Introduction

Healthcare priority-setting is a morally complex task. When resources are scarce, using them for one purpose precludes using them for another, resulting in some individuals or groups in society gaining while others lose. As such, healthcare priority-setting is also a politically complex task, requiring decision-makers to balance the interests of different groups, all of whom have a potential claim on shared resources.

In England and Wales, this task falls largely on the National Institute for Health and Care Excellence (NICE), an independent public body that advises the National Health Service (NHS) on its adoption of new health technologies. Established in 1999 on the back of public concern about the state of the NHS, NICE appeared to offer a “technocratic fix” to the intensifying political debate about the need for healthcare rationing [1]. NICE’s unusual longevity is evidence of its success in defusing this debate and in allowing politicians to remain at an expedient remove from the thorny question of how NHS funds should be spent.

NICE’s establishment gave it the necessary authority to fulfil its intended role [2]. But NICE has good reason to want to demonstrate that its decisions are morally, as well as legally and politically, legitimate. As a public institution, NICE is responsible for serving the public good through the promotion of health, the pursuit or protection of which holds primary moral importance [3–6]. NICE therefore has a *prima facie* reason to avoid acting in ways that are detrimental to population health. Social justice also compels it to ensure that the interests of the powerful do not come at a cost to the health and wellbeing of others [7]. On a practical level, priority-setting decisions can encounter strong societal resistance if they are considered unjust [8].

In seeking to demonstrate its moral legitimacy, NICE has long communicated the ‘social value judgements’ or ‘principles’ underlying its work [9–11]. Historically, these have centred on the need to balance the costs and benefits of adopting any new technology (its principle of cost-effectiveness) and on NICE’s use of a fair decision-making procedure, defined as one that meets the requirements of Daniels and Sabin’s ‘accountability for reasonableness’ (AfR) framework [12]. AfR has been widely adopted by healthcare priority-setters since the late 1990s [13] but, as others have observed, while a fair procedure might enhance legitimacy, it is not sufficient to ensure that decisions are substantively just [14–17]. Attempting to justify priority-setting purely with reference to substantive principles is, however, also problematic. Society holds diverse views about how shared resources should be distributed and many potential considerations – including the relevance of a technology’s cost-effectiveness – are the subject of reasonable disagreement [12, 18]. Any attempt to independently ground justification on substantive principles is therefore vulnerable to challenge from those who would simply argue that those principles are not correct.^1^ Even where there is public consensus about the appropriate principles, this does not guarantee that these principles are morally justified [28]. Neither substantive nor procedural approaches are, therefore, by themselves adequate to morally evaluate a priority-setter’s approach.

A complementary strategy is to base this evaluation on the overall coherence of a priority-setter’s moral system. Coherentist theories of moral justification are well established [29] and are grounded on the notion that, in the words of John Rawls, “a conception of justice cannot be deduced from self-evident premises or conditions on principles; instead, its justification is a matter of the mutual support of many considerations, of everything fitting together into one coherent view” [30]. This ‘fitting together’ is achieved through the method of reflective equilibrium: a process in which “we start with our existing ethical beliefs about cases and principles, weed out those that are thought to be unreliable, and then adjust the remaining set in order to make it as coherent as possible” [31].^2^ One route to morally evaluating a priority-setter’s actions is therefore to identify its various ethical beliefs and empirically assess the extent to which these ‘fit together’ to form a stable equilibrium [32].

Establishing equilibrium across the relatively narrow realm of priority-setting policy and practice falls short of the wide reflective equilibrium (WRE) that Rawls strove towards. Unlike WRE, narrow reflective equilibrium (NRE) generally does not defend the credibility of its content with reference to relevant background theories and wider perspectives and it is therefore acknowledged that NRE carries less justificatory power than its wide counterpart [33, 34]. However, on the view that moral justification is a matter of degree, establishing NRE is nevertheless a worthwhile aim and would represent a significant achievement that would bolster a priority-setter’s claim to moral legitimacy. Unlike the more aspirational WRE [30] establishing a relatively narrow equilibrium across a priority-setter’s own policy and practice would also seem to be a feasible goal for an institution with the power to shape its own approach.^3^

We do not suggest that this type of equilibrium is by itself sufficient for moral justification; we accept in principle that a priority-setter can act in ways both coherent and immoral. We also accept that the dynamic nature of any equilibrium will likely give rise to instances of transitory dissonance, as novel scenarios necessitate reflection on – and modification to – previously accepted policy and/or practice. But we argue that coherence carries justificatory power and that our confidence in any claim for moral justification should be lessened if substantial dissonance can be shown to persist within a priority-setter’s moral system.^4^We therefore propose that the empirical examination of coherence is a useful tool for moral evaluation and that, like the proverbial canary in the coalmine, the inability to fully establish NRE across a priority-setter’s policy and practice (or within either one of these domains) might act as a generalised warning that something is awry in a priority-setter's moral system [32].

Taking this theoretical position as its starting point, this study empirically examines the coherence of NICE technology appraisal policy by systematically identifying the normative commitments it contains and by attempting to bring these into equilibrium. Its aim is to support the formation of evidence-based conclusions about the extent to which NICE’s approach can be defended as morally justified and to identify potential sources of dissonance that may undermine this claim. It is our hope that this might motivate further discussion of how NICE’s approach might be modified to improve its coherence and moral legitimacy.

## Methods

The study is grounded in the theory, methods and standards of empirical bioethics: a discipline that attempts to address normative questions through the integration of empirical methods with ethical argument [37, 38]. This study uses thematic analysis as its empirical method [39] and a modified version of normative empirical reflective equilibrium (NE-RE) as the means of integration [40].

The aim of the study is to assess the coherence of NICE technology appraisal policy as applied to pharmaceuticals, as of December 2021.^5^Its scope does not extend to the practice of NICE technology appraisal; that is, to the case-based judgements made by appraisal committees in their consideration of specific technologies. Though we acknowledge that practice comprises an essential element of NICE’s overall approach and that the most reliable conclusions about coherence are those drawn from an analysis of both policy and practice, the complexity of this task and of NICE’s approach is such that it would not be feasible to report the findings of such an analysis in a single paper. Our work is therefore split into two stages. The first stage, reported in this paper, examines the coherence of NICE policy and allows us to draw conclusions about the extent to which NICE’s formal approach (that is, the approach specified on paper) is morally justified. The second stage, which is currently underway, examines the conclusions reached by appraisal committees in specific cases and will allow us to draw more comprehensive conclusions about the extent to which NICE’s approach as a whole – incorporating both policy and practice – can be brought into equilibrium. The results of the second stage of work will be reported in due course.

Analysis for this stage of the study was conducted across twenty policy documents spanning two NICE workstreams: the core technology appraisal (TA) programme, established in 1999, and the Highly Specialised Technologies (HST) programme for the appraisal of drugs for very rare diseases, established in 2013. These workstreams both focus on pharmaceuticals and are the only NICE programmes to carry a funding mandate: a statutory requirement for the NHS to fund the technologies recommended [42]. Analysed documents include formal accounts of NICE’s processes and methods, public articulations of NICE’s role and approach and various technical updates, addenda and position statements (Appendix 1). Previous work drawing on a similar data set had already highlighted changes to NICE’s methods over time and inconsistencies between NICE’s most recent articulation of its normative approach (*Our Principles*, hereafter *Principles*) and its current methods [43, 44]. However, this previous work did not comprise an attempt to comprehensively evaluate the coherence of NICE policy at a given point in time. A de novo analysis was therefore conducted for the current study.

An initial pilot analysis was conducted on NICE’s 2014 Clinical Guidelines Manual, a document that was outside the study’s scope but was similar enough to the documents of interest to allow coders to identify any flaws in the planned method, ensure consistent classification of deductive codes and identify an initial set of subcodes. Documents were coded according to accepted techniques of thematic analysis [39] – a method that previous studies have shown to be effective for exploring similar research questions [43, 45–48].

**Fig. 1:**
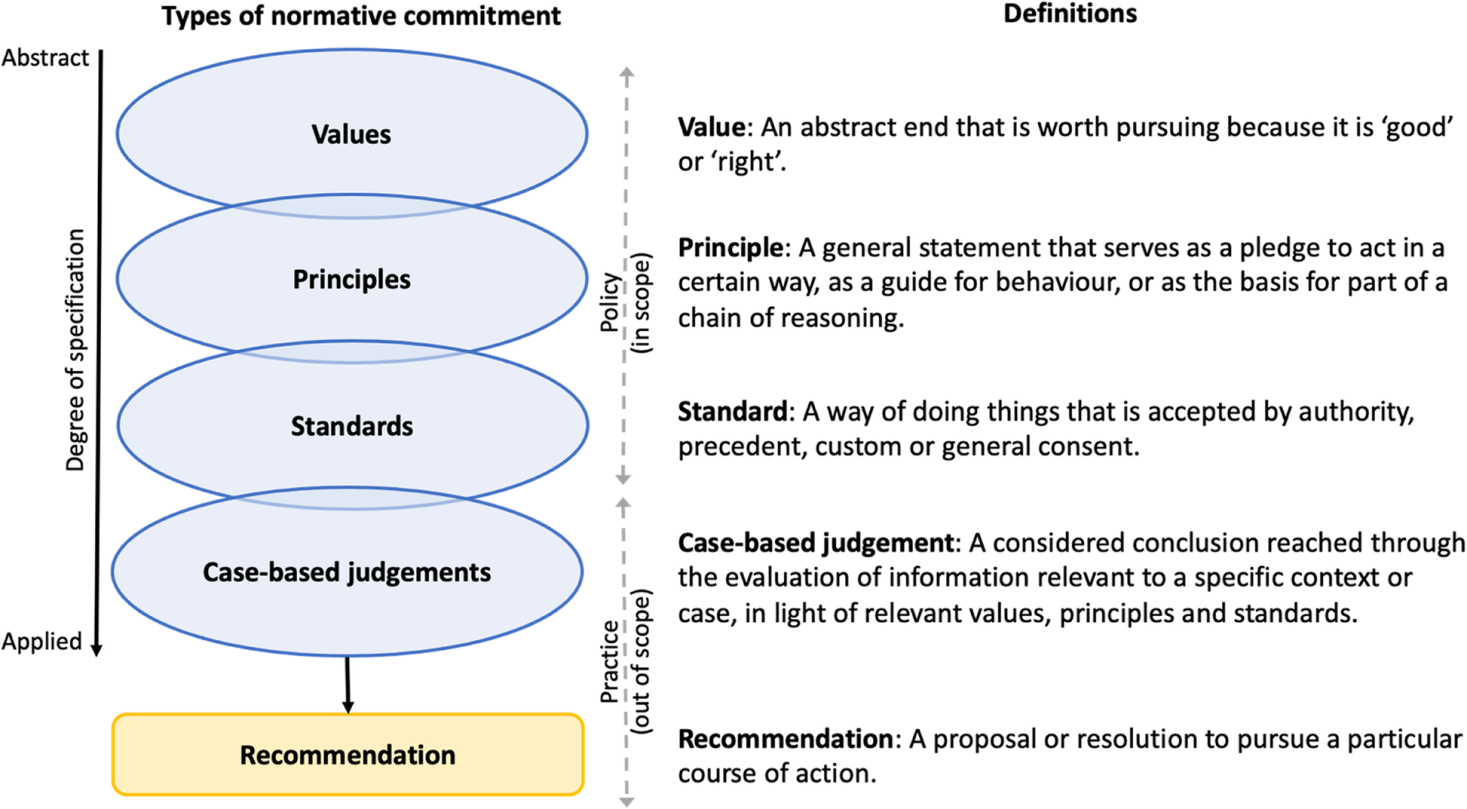
A framework for considering normative content in health technology assessment (Figure based on material derived from Charlton et al. (2023). The original source is an open access article, distributed under the terms of the Creative Commons Attribution licence, which permits unrestricted re-use, distribution and reproduction, provided the original article is properly cited.)

Thematic analysis proceeded via a mixed deductive-inductive approach. Deductive codes were primarily defined according to a conceptual framework that classifies normative content as either substantive (relating to what decisions are made and why) or procedural (relating to how decisions are made) and as either a value, principle, standard or case-based judgement, based on degree of specification [49]^6^ (Fig. 1). Further subcodes were carried over from the pilot analysis or were identified inductively based on their content, before being gradually refined through an iterative process. For example, the statement “People have the right to make informed choices about the care they receive” [11] was first deductively coded as a substantive principle and then further inductively sub-coded as ‘Individual choice’. This approach enabled the large amount of normative content embedded within hundreds of pages of NICE policy documentation to be organised in a way that allowed for coherence to be systematically assessed.

Initial coding was conducted by VC with duplicate coding conducted by MD across approximately 20% of the material, purposively sampled such that duplicate coding concentrated on material that was rich in normative content. Any disagreements were discussed and resolved. Codes were then collated, reviewed and refined, and then used to develop a detailed map of relations between the normative commitments embedded in NICE policy. This map was itself reviewed and refined and an attempt was made to bring as many components as possible into equilibrium. For the purpose of reporting, normative commitments which were found to be related through multiple mutually supportive relations were collected into ‘clusters’, while commitments that did not align with these clusters were identified as potential sources of dissonance.

Both NE-RE as originally conceived and our modification of the method are grounded on the theoretical claim that coherence can act as a source of moral justification. However, while NE-RE aims to employ empirical data alongside ethical analysis to “arrive at a justified view on the ethical acceptability of real-life ethical dilemmas” [50], our modified method aims to evaluate the level of coherence pertaining across an existing set of normative commitments. That is, while NE-RE seeks to generate a set of morally justifiable beliefs, our modified approach seeks to evaluate an existing set of beliefs in order to establish the extent to which they might be justified. Our method also differs from NE-RE in its source of empirical data: while NE-RE typically collects data on public perspectives and uses these as a source of potentially justifiable moral beliefs (*ibid.*), our approach collects data on a single institution’s moral system (although, in NICE’s case, this system is itself multifaceted and has been developed through a collaborative process that reflects the views of many internal and external stakeholders as well as public perspectives).

Despite these differences, our method has much in common with NE-RE in terms of both its theoretical grounding and its reliance on empirical data as a basis for ethical analysis. As such, we followed NE-RE’s approach in understanding coherence to be a function of the strength of the inferential connections that exist between elements of a moral system, with positive relations contributing to coherence and negative relations undermining it [40, 50]. In considering whether it was possible to establish equilibrium, we paid attention to both positive inferential relations (that is, sets of mutually supportive commitments) and sources of dissonance, which we identified as instances in which two or more normative commitments appeared to be connected via negative inferential relations. Commitments that were unrelated to others via either positive or negative relations were considered to neither contribute to nor undermine coherence.^7^

Although the analysis covered both substantive and procedural aspects of NICE policy, for reasons of space this paper focuses on the former, with considerations of the latter limited to those instances in which there is an especially close relationship between substance and procedure. We intend to report findings with respect to NICE’s procedural approach separately at some point in the future.

This study did not require ethical approval. A copy of the extensive analytical guide is available on request.

## Findings

### Overview

In total, the study identified 7 values, 63 principles and 176 standards from the substantive normative content of NICE technology appraisal policy (Appendix 2). Much of this content centres around a collection of values – fairness, reasonableness, lawfulness, legitimacy and health – that are highly coherent and provide a strong foundation for a morally justified approach. However, these values – hereafter referred to as the ‘primary cluster’ – are, on occasion, specified in ways that introduce dissonance and undermine coherence across this collection of normative commitments.

**Fig. 2:**
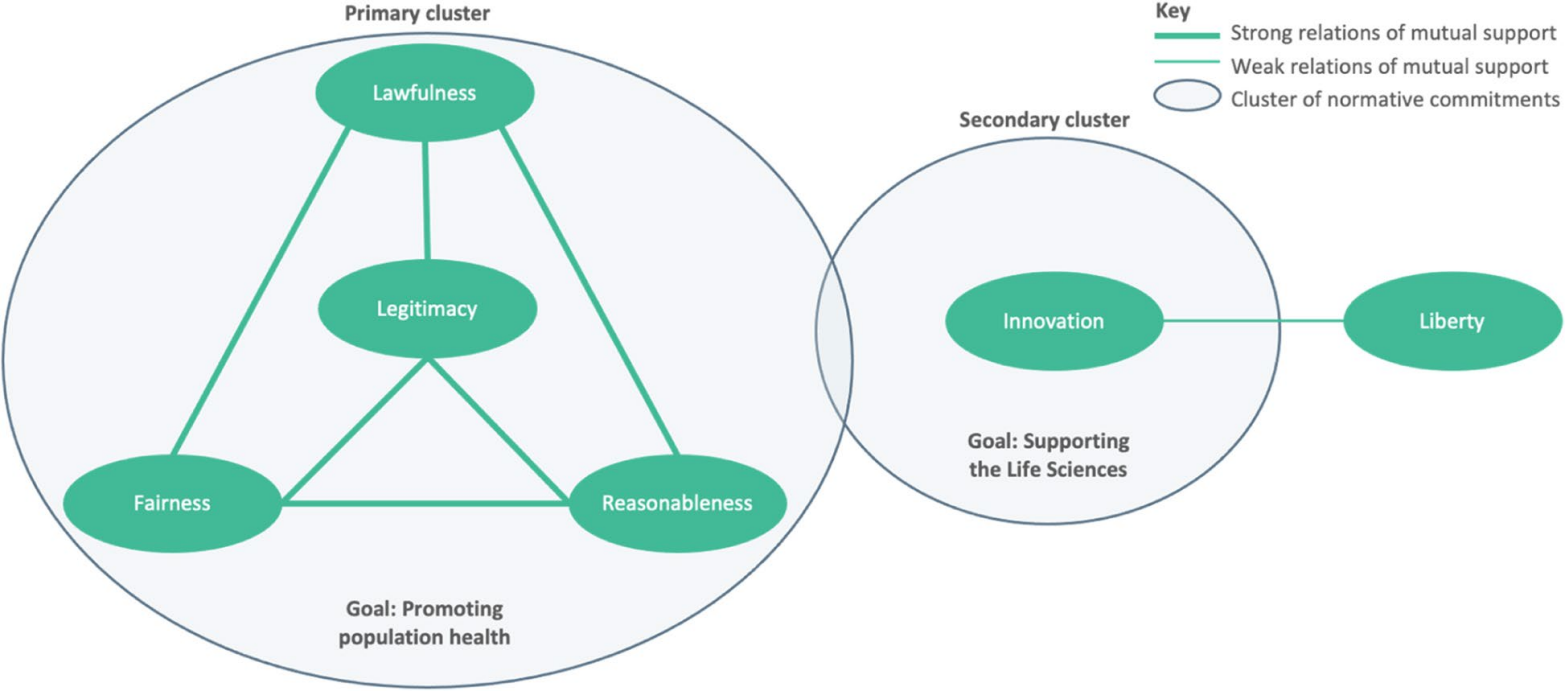
Map of relations between identified substantive values

In addition to the primary cluster, a second cluster of commitments centred around the value of innovation was also identified. Although these two clusters are linked through some supportive relations, there exists substantial potential for dissonance between them. A final value, liberty, aligns weakly with this secondary cluster (Fig. 2).

**Fig. 3:**
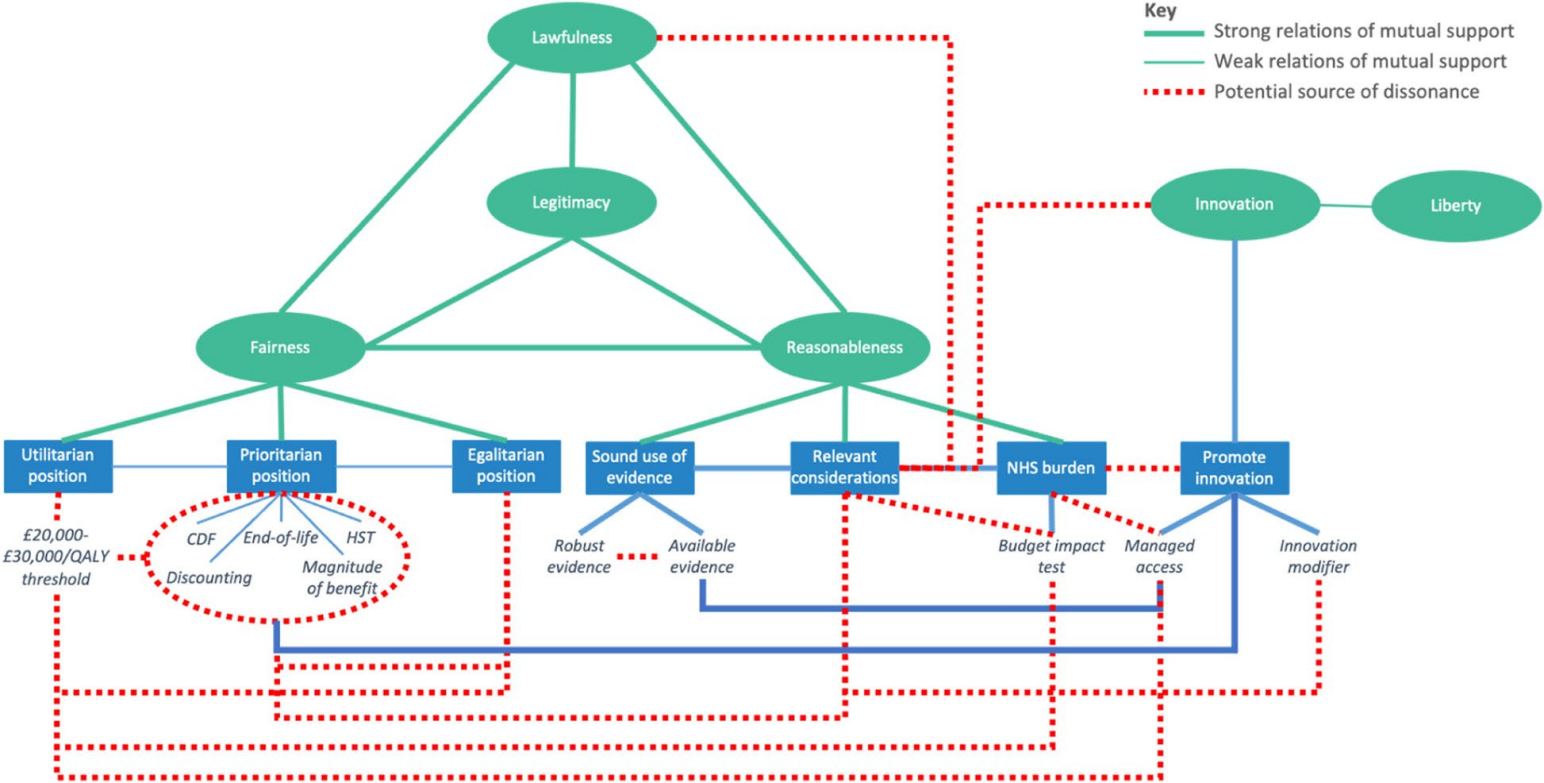
Identified sources of potential dissonance in NICE policy

The following sections describe each of these values and examine the key positive and negative relations that exist between them, and between the principles and standards through which they are specified (Fig. 3).

### The primary cluster

The primary cluster is centred on the values of fairness, reasonableness, lawfulness, legitimacy and health itself. Strong relations of mutual support exist between each of these values, but legitimacy plays a particularly central role in generating coherence across this cluster.

Legitimacy is generally defined as the value associated with having the right to govern, or with having one’s decisions attract obedience and respect [52]. Although NICE makes relatively few direct references to legitimacy, policy recognises that “NICE’s guidance can have a significant impact on people’s lives” [11] and that this requires NICE to act in ways that can be justified with reference to other values. One such value is fairness, with NICE committing to conducting its work in a way that is procedurally fair, as defined by the AfR framework. This framework is itself conceptually grounded on the need for healthcare priority-setters to demonstrate legitimacy, thus, according to NICE’s *Social Value Judgements* (hereafter *SVJ)*, fulfilment of these requirements gives “legitimacy to NICE guidance” and allows it to meet its “legal and moral obligations to the people it serves” [9]. *Principles*, which superseded *SVJ* in 2020, alludes less directly to the value of legitimacy, but similarly recognises that NICE’s advice must be seen as “valid[…]” and “credibl[e]” if it is to be accepted by society and by the medical establishment tasked with implementing it [11].

The AfR framework also directly connects legitimacy to the notion of reasonableness and the idea that priority-setting decisions should be based on a “detailed consideration of the evidence” and reasons that fair-minded people would consider to be relevant [11].^8^ Though forming part of a procedural framework, this notion of relevance is inherently substantive (and, as will shortly be discussed, is not always consistently specified across NICE policy). NICE’s pledge “to treat people fairly” [53] is also presented as a substantive commitment that exists independent of legitimacy and which constitutes a quasi-legal requirement under the terms of the NHS Constitution [11], which also includes a commitment to fairness [54]. This respect for the NHS Constitution is indicative of a wider commitment to lawfulness which further contributes to NICE’s legitimacy and takes formal precedence over other considerations.

The central position that fairness, reasonableness and lawfulness occupy in NICE policy is also reflected in the design of its appeals process which, according to Daniels and Sabin, confers further accountability by allowing decisions to be formally challenged [12]. Grounds for appeal closely reflect these core values and are limited to claims that NICE has acted unlawfully, unreasonably or in ways that are procedurally unfair [55]. Thus, much of NICE’s moral system can be seen to cohere around these highly plausible values, providing a strong basis for morally justified decision-making. Underlying this system is a commitment to the value of health itself and to the goal of helping “health and public health professionals […] to deliver the best possible care within the resources available” [56].

However, despite the positive relations that exist between these key values, sources of dissonance exist between the principles and standards through which they are specified. It is to these more detailed aspects of NICE policy that we now turn.

### Fairness

Central to NICE policy is a complex substantive conception of what constitutes a “fair and equitable” allocation of resources [11]. This conception incorporates commitments of a utilitarian, prioritarian and egalitarian nature and, though these commitments could potentially be balanced in coherent ways, their current specification gives rise to several sources of dissonance.

### The utilitarian view: the relevance of opportunity cost

NICE’s main substantive claim is that it bases its recommendations on “an assessment of population benefits and value for money” [11]. This indicates a broadly utilitarian view of fairness which reflects a moral concern for the absolute amount of health that can be generated from a given healthcare budget.

This claim is articulated in a variety of different ways. On occasion, NICE suggests that the objective of the NHS is to “maximise” population health and that its recommendations are intended to support this goal [53]. But though NICE’s approach acknowledges the importance of allocative efficiency, neither it, nor the NHS, actually adopts a maximising approach, because it does not consider the entire NHS offering. Rather, NICE demonstrates its concern for opportunity cost – that is, the benefits foregone when a choice is made to employ resources in one way rather than another – by having regard for “the broad balance between the benefits and costs” associated with the adoption of specific technologies [11]. This regard is statutorily mandated and is specified through NICE’s principle of cost-effectiveness, which asserts that: i) a technology should only be recommended if it is deemed cost-effective, and ii) a technology should generally be deemed cost-effective only if “its health benefits are greater than the opportunity costs of programmes displaced to fund the new technology” [53].^9^

This principle is put into practice through various standards that specify how a technology’s likely benefits and costs should be assessed and balanced. These include the definition of a general ‘threshold’ of £20,000-£30,000 per quality-adjusted life-year (QALY) beyond which a technology will not usually be deemed cost-effective [53]. However, this principle is deliberately formulated to allow flexibility and does not exclude the possibility that this threshold might be exceeded or that QALYs might be valued differently when they are experienced by particular groups. Thus, though the default is to value all health equally, appraisal committees have “discretion to consider a different equity position and may do so in certain circumstances” *(ibid.*). This allows NICE to maintain equilibrium between its formal principle of cost-effectiveness and other non-utilitarian principles which recognise that “in the interests of fairness, the needs of particular groups may override those of the broader population” [11].

Notwithstanding the potential coherence of this general approach, NICE’s standard of generally adopting a threshold of £20,000-£30,000/QALY appears inconsistent with its commitment to cost-effectiveness because, according to the best available empirical evidence, the annual marginal cost per QALY in the NHS is actually much lower than this threshold implies (at between around £5,000 and £10,000/QALY [58–60]). This suggests that “guidance issued by NICE is likely to do more harm than good, reducing health outcomes overall for the NHS” [58]. Thus, while the principle of cost-effectiveness could potentially exist in equilibrium with other elements of the primary cluster, there appears to be dissonance between this principle and the standard through which it is currently specified.

### The prioritarian view: not all health is equal

Under a parallel conception of fairness, NICE addresses the morally distinct needs of particular groups through principles and standards that can be broadly classified as prioritarian: a view of fairness that values benefits more highly when they are experienced by those suffering from disadvantage [61]. This view is reflected in NICE’s statutory requirement to consider not just a technology’s cost-effectiveness, but also “the degree of need” of NHS users [62].

Defined simply as thus, NICE’s commitment to both prioritarian and utilitarian views can be easily brought into equilibrium: cost-effectiveness will generally be a major consideration, but committees may value QALYs experienced by high-need groups more generously in assessing a technology’s benefits. However, dissonance arises from the specification of this view through several overlapping ‘prioritisation standards’ contained within NICE policy:

- a) Exceptional discounting. NICE’s methods generally specify that, when estimating a technology’s cost-effectiveness, both the costs and benefits associated with its adoption should be discounted at an annual rate of 3.5% [53]. This is standard practice in economic evaluation and is intended to reflect the ‘time value’ of money.^10^ However, since 2011, when a technology provides very significant long-term health benefits to patients who would otherwise die or experience significant health loss, NICE’s methods have allowed a reduced discount rate of 1.5% to be applied, increasing the value of a technology’s long-term (health) benefits relative to its short-term (financial) costs [53]. The effect is to improve a technology’s apparent cost-effectiveness to the benefit of severely ill young- to middle-aged patients whose needs are addressed through highly effective but expensive treatments.^11^
- b) Increased threshold for end-of-life technologies. A technology’s likelihood of being deemed cost-effective can also be improved through application of the ‘end-of-life’ standard. Applied specifically to technologies that treat conditions associated with a life-expectancy of less than two years and which are expected to extend life by at least three months, this standard allows committees to give “greater weight to QALYs achieved in the later stages of terminal diseases”, up to an effective threshold of £50,000/QALY [53, 65]. In its first iteration, application of this standard was limited to technologies indicated for “small patient populations” – classified as a maximum of 7000 patients in England [53]. Evidence suggests that this standard has only ever been successfully applied to cancer drugs [66].
- c) Recommendation via the Cancer Drugs Fund. When the available evidence about a technology is weak or indicates that the technology is not likely to meet the relevant threshold, concern for cost-effectiveness implies that it should usually be rejected. However, if that technology is a cancer drug that has shown “plausible potential” for clinical- and cost-effectiveness, then it may be provisionally recommended on the condition that further evidence continues to be collected [65]. Such technologies are paid for via the NHS’s dedicated ‘Cancer Drugs Fund’ (CDF), which NICE has operated since 2016.
- d) Increased threshold for highly specialised technologies (HSTs). NICE’s HST programme, established in 2013, considers a small group of ultra-expensive technologies that are exclusively indicated for very rare and severe conditions [67]. It prioritises patients suffering from such conditions through use of an increased cost-effectiveness threshold of £100,000/QALY.
- e) Magnitude of benefit weighting for HSTs. HSTs also benefit from the additional weighting of QALYs generated by especially effective treatments. This amounts to a further uplift in the HST threshold to between £100,000/QALY and £300,000/QALY, depending on the total number of QALYs that a patient is likely to gain through access to a new drug [67]

These standards are all, individually, potentially consistent with NICE’s concern for both cost-effectiveness and clinical need and there may be good ethical reasons for wanting to prioritise each of these groups. But these standards each interpret need differently and they are not unified by any well-defined and morally plausible principle of prioritisation. This gives rise to several sources of dissonance and prevents these standards from being simultaneously incorporated into the primary cluster in a way that is coherent.

One source of dissonance arises as a function of the algorithmic approach necessarily used in determining whether particular patient groups meet the criteria specified by these standards, leading to clinically similar patients falling on either side of an arbitrary line and consequently receiving substantially different consideration. For example, ultra-rare diseases treated with HSTs experience far greater priority than merely rare diseases treated with non-HSTs, while a cancer indication for which average life-expectancy is 23 months would, according to strict application of the end-of-life standard, be treated much more favourably than a similar indication with life-expectancy of 25 months.^12^ Such ‘threshold effects’ do not align well with egalitarian principles of fairness, which are grounded on notions of equality (see below), and, given the clinical similarities that exist between these groups, they also fail to cohere with a general prioritarian desire to preferentially help those most in need.

The potential unfairness arising from this algorithmic approach is amplified by the ‘ultra-prioritisation’ of certain patients due to different prioritisation standards targeting overlapping groups. Technologies indicated for young adults suffering from very rare, very severe diseases, for example, may benefit from application of a reduced discount rate (standard a) above), an enhanced HST threshold (standard d)) and HST QALY weighting (standard e)), leading to their being assessed according to a comparative threshold of £300,000/QALY or more. In contrast, a technology indicated for slightly older adults suffering from a similarly serious but more common condition may meet none of the criteria for prioritisation and would consequently be assessed according to a threshold ten to fifteen times lower. Similarly, patients suffering with terminal cancer may benefit from both application of the end-of-life standard (standard b)) and recommendation based on uncertain evidence via the CDF (standard c)), while patients suffering from other conditions with marginally better prognoses may benefit from neither. Such idiosyncrasies generate morally dubious outcomes that undermine coherence and give cause to question on what grounds the (unstated and somewhat ambiguous) principles shaping these standards might be defended.

These standards are also occasionally inconsistent with other aspects of NICE’s approach. NICE generally takes the view that “mere survival is an insufficient measure of benefit” when assessing a technology’s value and that “the expected quality of life years gained also needs to be considered” [9]. Its preferred measure of health is therefore the QALY, which considers both the quantity- and quality- of any life-extension arising from a technology’s use. In applying the end-of-life standard (standard b)), however, NICE advises its committees to assume that any life-extension “is experienced at the full quality-of-life anticipated for a healthy individual of the same age” [53]. Given the significant symptoms experienced by many terminally ill patients – and the unpleasant side-effects associated with many life-extending drugs – this assumption is empirically implausible. Rather, it seems to reflect the normative view that, in these circumstances, ‘mere survival’ is all that counts. This is a deviation from NICE’s usual position and is inconsistent with NICE’s treatment of other terminally ill patients whose health is considered to be a function of both quantity and quality.

Other prioritarian standards are potentially incompatible with substantive principles historically endorsed by NICE in *SVJ*. The use of a lower discount rate for technologies substantially benefitting younger people, for example, could be considered to conflict with the principle that patients’ access to technologies should not be restricted “because of their age” [9].^13^ Likewise the weighting of QALYs based on the total magnitude of a technology’s benefit in the HST programme, which is necessarily limited by a person’s natural life expectancy and disadvantages older patients. Equally, the existence and operation of the HST programme is difficult to reconcile with the principle that drugs for rare diseases should be evaluated “in the same way as any other treatment” and arguably does not accord with NICE’s claim that it “cannot apply the ‘rule of rescue’” – a reference to the moral inclination to want to help an identifiable person whose life is in danger, whatever the cost [9, 11].^14^

Some of this dissonance has been superficially mitigated by the publication of *Principles,* which NICE variously describes as ‘superseding’ or ‘replacing’ *SVJ* and which omits reference to either age or rarity [11]. However, the precise status of *SVJ* in NICE’s evolving approach is ambiguous^15^ and it is unclear to what extent appraisal committees are expected to follow *SVJ*’s advice in practice. Moreover, NICE’s decision not to acknowledge or justify its basis for prioritising specific groups, in *Principles* or any other current articulation of its approach, is inconsistent with its commitment to AfR and to ensuring that its reasons for decision-making are open to scrutiny.^16^ Thus, while there may be good ethical grounds for NICE’s prioritisation standards – and perhaps even a unifying principle that brings them into equilibrium with one another and with other elements of the primary cluster – NICE’s failure to properly acknowledge and justify such grounds is itself inconsistent with its stated values, undermining coherence.

### The egalitarian view: all principle, no substance

Egalitarianism exists in many forms, but all are grounded on the idea that people are equal and deserve equal rights and opportunities [30, 61]. In specifying this idea in a way that aligns with utilitarian conceptions of fairness, NICE has historically focused on the need to avoid unfair discrimination in limiting its recommendations to particular groups, in order to avoid the inequitable outcomes that might otherwise arise from a desire to allocate resources efficiently. Thus, *SVJ* states that recommendations can only be restricted by group “in certain circumstances” and specifies that “NICE should not recommend interventions on the basis of individuals’ income, social class or position in life” [9].

More recently, egalitarian principles have come to play a more central role in NICE policy, with NICE now actively aiming to “reduce health inequalities” through its work [11]. According to *Principles*, consideration of “socioeconomic factors and the circumstances of certain groups of people” is now actively encouraged and recommendations may be targeted at such groups if this helps to “reduce and not increase identified health inequalities” [11]. This advice is designed to cohere with NICE’s obligations under the UK Equality Act 2010, in support of NICE’s commitment to lawfulness. However, it is not specified how the principle of cost-effectiveness should be balanced against strategies intended to “improve population health as a whole, while offering particular benefit to the most disadvantaged” [11]. It is also unclear how NICE’s concern for socioeconomic considerations might interact with its prioritarian conception of fairness, which currently defines disadvantage according to clinical need rather than socioeconomic factors. Thus, though NICE’s egalitarian goals are clear, the means by which these might be coherently balanced against other goals remains unspecified.

NICE’s commitment to reducing health inequality may also act as a source of dissonance given NICE’s current definition of cost-effectiveness. Empirical evidence suggests that, at a cost-effectiveness threshold of £20,000-£30,000/QALY, health outcomes for the NHS as a whole will be reduced as a result of NICE’s advice [58–60] and that this health loss will fall disproportionately on socially deprived groups [69]. It is therefore questionable whether NICE’s commitment to equality, as currently specified, can be implemented in a way that fully coheres with other aspects of its approach.

### Asymmetric prioritisation: unequal treatment of equal health?

Tensions naturally arise between NICE’s substantive views of fairness. But these are not insurmountable, and coherence could likely be materially improved through relatively small changes to ways that these views are formally specified and balanced. There is, however, one aspect of NICE’s approach that poses a more substantial challenge to coherence in general and fairness in particular.

The source of this challenge lies in the asymmetry that exists between NICE’s treatment of those groups whom it prioritises through the standards discussed above and the treatment of similar groups whose needs are addressed elsewhere in the NHS. Take, for example, patients suffering from a particular type of late-stage cancer. Under NICE’s current approach, application of the end-of-life standard will mean that health gains generated by treating these patients with a life-extending drug will be valued more highly than other types of health gain, facilitating the drug’s recommendation above the usual cost-effectiveness threshold. But in adopting this technology the NHS will need to divert resources away from other potential uses, some of which may have benefitted similar (or even the same) patients. To fund access to the new drug, it may be necessary to divert resources away from palliative care wards, for example, or to close surgical lists leading to increased waiting times for other late-stage cancer patients. If the health of late-stage cancer patients is deemed especially valuable – as NICE’s approach suggests – then logic requires that these health losses also be valued more highly when they are suffered by such patients, implying an increase in the opportunity cost associated with adopting the new drug. As others have pointed out, NICE’s approach does not currently take account of this increase and is therefore logically inconsistent [70, 71].

It may be that this apparent inconsistency can be justified with reference to values other than fairness, such as innovation (on which more later). However, such justification would not resolve the negative relation that seems to exist between this unequal treatment of clinically similar patients and all three of NICE’s conceptions of fairness. Moreover, NICE’s failure to acknowledge this challenge or offer any justification for why this outcome should be accepted as fair itself undermines its commitment to procedural fairness. Thus, asymmetric prioritisation appears to seriously undermine the coherence of NICE’s approach and raises significant questions about the extent to which NICE policy can be defended as morally justified.

### Reasonableness

Alongside references to fairness, NICE highlights the importance of reasonableness in supporting the legitimacy of its decisions.

Reasonableness can generally be understood as the value derived from acting according to reason – it does not necessarily require that these reasons be made available to others [72]. However, in NICE’s approach transparency is often presented as a necessary corollary to reasonableness due to AfR’s requirement that the reasons for decision-making be made public [9]. Implicit in NICE’s pledge to make “a clear case” for adopting certain technologies [56] is therefore a commitment to both acting according to reason and to communicating these reasons openly.

NICE does not explicitly set out how it ensures that its decisions are reasonable. However, three underlying principles can be inferred from policy: i) that decisions should reflect the sound interpretation of evidence, ii) that decisions should reflect the appropriate treatment of relevant considerations, and iii) that decisions should not be so demanding as to place an excessive burden on the health service. These principles – and their implications for coherence – are considered in turn.

### Decisions should reflect the sound interpretation of evidence

NICE’s public identity centres on its status as an “evidence-based” decision-maker [56]. Use of evidence is therefore central to NICE’s definition of reasonableness and several standards – including the requirement for evidence to be systematically reviewed, the involvement of technical experts and the use of a standardised ‘reference case’ for economic analysis – exist to ensure that evidence is gathered, used and interpreted appropriately. More ambiguous is NICE’s specification of what constitutes adequate evidence and what reasonableness requires when the best available evidence is weak.

Much of NICE policy suggests that reasonable decisions, by definition, are informed by good evidence. The *Methods Guide*, for example, explicitly states that if NICE’s decisions are to be “appropriate and robust”, it is “essential that the evidence and analysis, and their interpretation, are of the highest standard” [53]. *Principles* echoes this position, underlining that “NICE’s guidance and standards are underpinned by evidence” and that, as such, “we need to ensure that this evidence is relevant, reliable and robust” [11]. Elsewhere, however, NICE acknowledges that although it “bases its decisions on the best available evidence […] this evidence is not always of good quality and is hardly ever complete” [9]. The implied claim that ‘evidence-based’ decisions are based on good evidence is thus at variance with the fact that the best available evidence may be “conflicting, insufficient or not robust” [11].

When the best available evidence is weak, NICE policy is inconsistent about whether it is reasonable to recommend a technology’s adoption. NICE claims that planned appraisals will not be taken forward if “appropriate” [73] or “adequate” [67] evidence is not available and it has long held as one of its key principles that “committees should not recommend an intervention if there is no evidence, or not enough evidence, on which to make a clear decision” [9, 11]. Indeed, NICE has described it as “the role of the Appraisal Committee not to recommend treatments if the benefits to patients are unproven” and committees are specifically advised that they should “be more cautious about recommending a technology when they are less certain” about its effects, particularly as its cost/QALY increases [53]. Taken together these statements strongly imply that, in the absence of good evidence, NICE adopts the conservative view that the distributive status quo should be maintained in preference to recommending a technology of uncertain value.

Other standards, however, are specifically designed to facilitate the adoption of technologies for which evidence is “absent, weak or uncertain” [53]. One option is for uncertain technologies to be made available “only in the context of research” (*ibid.*) – a situation that carries little financial burden for the NHS and is therefore broadly consistent with the concern for opportunity cost that underlies a conservative preference for the distributive status quo. Another option, however, allows that when there is “high uncertainty in the evidence” about a new technology, that technology may be provisionally adopted – at cost to the NHS – based on its “plausible potential” for clinical- and cost-effectiveness [74]. Such managed access arrangements – which include the CDF – reflect a ‘pro-innovation’ view in which, in the absence of good evidence, preference is generally given to the adoption of a new technology, providing it shows sufficient promise.

There are obvious semantic inconsistencies between some of these statements which serve to undermine coherence, in part by eroding transparency. It is difficult to reconcile NICE’s claim that its decisions are based on evidence that is “relevant, reliable and robust” [11], for example, with its willingness to routinely recommend technologies for which the available evidence is “absent, weak or uncertain” [53]. But a more serious issue is the potential dissonance that exists between these two approaches to managing uncertainty: the conservative approach and the pro-innovation approach. The high burden of evidence placed on technologies seeking to displace existing interventions under the conservative approach protects the interests of general NHS users by ensuring that opportunity cost will only be imposed if a new technology’s benefits are likely to justify this trade-off. In contrast, the pro-innovation strategy of supporting the adoption of highly uncertain technologies whose benefits are not (yet) assured protects the interests of those patients (and manufacturers) who stand to benefit from a new technology’s adoption.^17^ These positions are not irreconcilable, but if coherence is to be maintained then the interests of these two groups must be very carefully balanced.

Much depends on the threshold at which committees are willing to recommend highly uncertain technologies and the opportunity costs that are therefore incurred. But though NICE advises its committees to exercise caution, policy does not specify what is an acceptable cost-effectiveness threshold in the context of uncertainty, allowing for the possibility of dissonant outcomes. Moreover, the commercially confidential nature of managed access arrangements means that the cost per QALY used in decision-making will generally not be disclosed, preventing public scrutiny and undermining procedural fairness.

### Decisions should reflect the appropriate treatment of relevant considerations

Although NICE’s conception of reasonableness rests (to varying degrees) on the use of evidence, it also acknowledges that appraisal committees “have to make judgements” about what a technology is worth [11]. If the outcome of appraisal is to be accepted as reasonable, it is therefore “important” that a committee can “explain what informs those judgements” (*ibid.*) and that those judgements reflect the appropriate treatment of relevant considerations.

NICE follows the AfR framework in defining relevance as grounds “that fair-minded people would agree are relevant in the particular context” [9]. This definition allows that relevant considerations may vary across cases and NICE accordingly highlights that committees have “discretion to consider those factors [they believe] are most appropriate to each appraisal” [53]. Nevertheless, NICE’s approach formalises the consideration of some factors such that their relevance (or irrelevance) is assumed; consideration of a technology’s clinical- and cost-effectiveness, for example, plays a part in all NICE decision-making.

This approach balances the potentially competing objectives of contextual sensitivity and substantive consistency in a way that supports the values of both fairness and reasonableness. However, there are discrepancies in how certain considerations are standardised that introduce dissonance, particularly in relation to HSTs. NICE generally does not treat patient population size as a relevant consideration and has traditionally taken the view that rare diseases should be evaluated “in the same way as any other treatment” [9]. But eligibility for consideration through the HST programme depends in large part on patient population size and leads to substantial prioritisation of some very rare conditions [75]. Within the HST programme further priority is automatically given to technologies that offer large QALY gains; another standard that is not echoed in the core appraisal programme, even for conditions of comparable severity. The HST programme is also unique in treating drug acquisition cost and the potential length of treatment as considerations relevant to topic selection, and in requiring the committee to “take into account what could be considered a reasonable cost for the medicine” when reaching a judgement about its suitability [67]. Looking beyond the HST programme, considerations that a fair-minded person could consider relevant – such as the effect of a technology’s use on economic productivity and a technology’s overall budget impact – are formally excluded from consideration, while other factors – such as whether a technology is indicated for cancer – are considered as a matter of policy despite their potentially questionable relevance [53].

While such considerations might feasibly be defended as relevant and therefore reasonable, NICE is not always open about their normative basis; it does not explain, for example, why magnitude of benefit is only formally considered in relation to HSTs or why cancer is granted exceptional status.^18^ This weakens its commitment to transparency and makes it difficult to establish equilibrium across standards that, on the face of it, appear to reflect an inconsistent view of what a fair-minded person would consider to be relevant considerations in healthcare priority-setting.

### Decisions should not place an excessive burden on the health service

When NICE makes a recommendation through its TA or HST programmes, the NHS is legally required to make funding available for the recommended technology, usually within a period of three months [42]. Even when a technology is cost-effective, this requirement constitutes a burden for a health service in which budgets are fully committed and new technologies generally must be funded, at least in part, by displacing other existing or planned activities. Several standards embedded within NICE’s approach indicate that it recognises this burden and conceives of reasonableness in a way that attempts to protect the NHS from excessive short-term demands, while continuing to respect the values of fairness and lawfulness.

This is most clearly reflected in the modification of NICE’s usual approach in response to technologies that have an extremely large budget impact. Budget impact is not generally deemed a relevant consideration in NICE decision-making and the *Methods Guide* specifically advises that this factor “does not determine the Appraisal Committee’s decision” in any individual case [53]. However, if the net annual cost of a technology’s adoption is expected to exceed £20 million, a formal process variation allows full adoption to be delayed for up to three years to allow the NHS time for operational planning and further opportunity for commercial negotiation [73, 76].

Although arguably a pragmatic response to operational challenges, this ‘affordability’ standard does not straightforwardly cohere with other principles of fairness and reasonableness. Deviation from the usual three-month implementation period is at variance with the goal of promoting population health insomuch as it may reduce efficiency by delaying the adoption of demonstrably cost-effective technologies. The use of a £20 million budget impact threshold is also potentially unfair in that it leads to similar patients being treated differently: whether due to patient population size or commercial pricing decisions, a patient whose treatment has a budget impact of £19 million per year will gain access within three months, while a patient whose treatment has a budget impact of £21 million may have to wait three years and could suffer substantial health loss as a result. If we accept that budget impact is not a morally relevant consideration, then this difference in outcome contravenes formal equality and arguably leads to unfair “numerical discrimination” [77–79]. On the other hand, given the very real financial and operational constraints that the NHS operates under, it could be argued that a decision that places excessive demands on the NHS today may harm its long-term sustainability and, as such, the health of future NHS users. A principle that protects the NHS from such demands might therefore support NICE’s goal of promoting population health, depending on how it is used and implemented.

It could also be argued that this principle provides indirect justification for NICE’s prioritisation of small patient groups. Technologies that are effective in treating severe disease, but which have a high cost per QALY, such as many HSTs and some cancer drugs, would impose a substantial burden on the NHS if they were indicated for large numbers of patients. But they impose a relatively small burden if they are indicated only for very rare diseases or for relatively uncommon indications (as in the first iteration of the end-of-life standard). Such technologies, considered individually, might therefore be deemed not to impose a significant burden on the NHS. This logic ignores, however, both the potential for unfair ‘numerical discrimination’ and the potentially large aggregate effect of adopting many such technologies over a period of time, particularly when these technologies are used as comparators in future appraisals, further inflating what is considered to be an acceptable cost per QALY. Concern for affordability therefore does not seem to resolve dissonance between NICE’s prioritisation standards and other elements of the primary cluster.

### Lawfulness

The last element of the primary cluster to be considered is lawfulness.

In modern democratic societies, lawfulness is an important component of legitimacy and legislation is generally designed to express the same values – fairness and reasonableness – that are at the heart of NICE’s approach [80, 81]. It is therefore uncontentious to suggest that these values are naturally well aligned. Coherence between these values is further enhanced by NICE’s statutory foundations, which partially specify its understanding of fairness [62], and in the formulation of NICE’s formal grounds for appeal, which largely mirror these values [55].

In addition, NICE explicitly states in policy its need to comply with several pieces of relevant legislation, including the Freedom of Information Act 2000, the Data Protection Act 2018, the Equality Act 2010 and international human rights law. It seems likely that this need for legal compliance has played an important historical role in NICE policy development and there are few instances in which NICE’s commitment to lawfulness is at variance with its other normative commitments. One possible exception is the Equality Act 2010, which closely defines what constitutes discrimination and potentially constrains NICE’s freedom to take into account the age of a particular patient population, despite the view of some fair-minded people that technologies indicated for children warrant prioritisation [82, 83].^19^ However, as previously discussed, NICE appears to have managed this potential for dissonance in part by declining to specify how it will act to promote equality and by leaving it to the discretion of its appraisal committees to ensure that coherence is maintained.

### The secondary cluster

The primary cluster is centred around a coherent set of mutually supportive values, specified in ways that occasionally introduce dissonance across the principles and standards that underpin them. The formulation of this cluster represents our best effort to establish equilibrium across NICE policy and incorporates most of its major elements. It does not, however, incorporate everything. Some elements of NICE’s approach do not share strong positive relations with the values at the heart of the primary cluster, but rather coalesce in the form of a second, smaller cluster that is similarly held together through a web of mutually supportive connections. This secondary cluster is centred on the value of innovation.

### Innovation

Ambiguous and value-laden, innovation is a complex and contentious concept. Nevertheless, NICE policy contains numerous references to the need to support innovation and regard for this principle forms part of NICE’s statutory function [53]. One interpretation of innovation locates its value wholly in its potential to promote population health – an interpretation that aligns well with the commitments contained within the primary cluster. An alternative interpretation, however, derives the value of innovation, at least partly, from its ability to contribute to progress in the commercial life sciences. This goal is by no means synonymous with that of promoting population health and this conception of innovation therefore introduces substantial potential for dissonance between the values at the heart of NICE’s approach.

In some documents, NICE appears to adopt the first interpretation, linking the value of innovation with the ability of new technologies to contribute “long-term benefits to the NHS” [9]. However, other specifications appear to decouple the value of innovation from its ability to generate direct health benefits and suggest that at least some of its value derives from other normative goals. In *Principles*, for example, NICE acknowledges that new technologies may not perform as well as expected and that “innovation does not necessarily lead to better outcomes than existing practice” [11]. Nevertheless, it commits NICE to supporting innovation “by encouraging interventions that provide substantial distinctive benefits that may not be captured by measuring health gain” (*ibid.*).

NICE is not clear in *Principles* what form these uncaptured benefits might take or why, in the context of healthcare priority-setting, they should be valued over and above a technology’s demonstrable health benefits. But other documents suggest that NICE’s concern for innovation may be motivated by political and economic (as well as health) goals. In a 2017 position statement, NICE states that though its “primary responsibility” is to promote population health, it also has a role to play in “contributing to UK economic growth” and “supporting a thriving life sciences sector” [85]. It must, it acknowledges, therefore sometimes adjudicate between “incompatible and competing influences” in deciding which technologies to recommend (*ibid*.). While NICE promises in this statement to manage this “tension” in ways that are “constructive” and “always oriented towards the best possible outcome for patients while ensuring value for money for the taxpayer” (*ibid*.), this commitment to maintaining equilibrium may not always be attainable and NICE’s approach, at times, seems to balance support for innovation against other goals in a way that undermines coherence.

This is demonstrated through two key standards which formalise NICE’s support for innovation. First, when considering a technology that does not meet the usual cost-effectiveness criteria, committees are required by the *Methods Guide* to consider a technology’s “innovative nature” in deciding whether it might nevertheless be recommended for adoption [53]. Specifically, committees are advised to consider any “health-related benefits” that may not have been accounted for in assessing an innovative technology’s value (*ibid.*). If the value of innovation is derived wholly from its impact on population health, however, this advice is illogical: demonstrable health benefits should already have been incorporated in the calculation of the technology’s cost/QALY and if other relevant health benefits have been overlooked, then an attempt should be made to capture them regardless of whether the technology in question is innovative. In asking committees to specifically consider benefits associated with a technology’s innovative nature, NICE seems to suggest that innovation is itself grounds for prioritisation, without offering any justification for this position.^20^

Second, NICE explicitly seeks to support innovation by using managed access arrangements to facilitate the adoption of uncertain technologies. As NICE recognises, innovative technologies are not necessarily clinically superior to current treatments and “if innovations come at an additional cost, they may divert resources away from existing practices that are better value for money” [11]. Managed access arrangements are thus framed as a way to “mitigate the risk” (*ibid.*) associated with such decision error by allowing commercial terms to be renegotiated – or recommendations reversed – if further data collection reveals a technology to be less beneficial than expected. However, the risk associated with adopting uncertain technologies remains substantial because, during the period of managed access, the NHS will inevitably incur opportunity cost. Theoretically, if a technology is adopted at a cost of £20,000-£30,000/QALY and delivers the anticipated benefits, then this opportunity cost will be offset by the benefits that the new technology delivers.^21^ In this scenario, equilibrium is just about maintained between NICE’s support for innovation and the principle of cost-effectiveness. But in proposing a managed access arrangement, NICE encourages manufacturers to focus on “the most compelling data” [85] and seeks to support them in putting forward the “best plausible case for the use of their product” [74]. Such a case will, by definition, present the maximum benefits that can plausibly be attributed to the technology’s use. It is therefore more likely than not that the actual benefits will be less than anticipated and, at a cost of £20,000-£30,000/QALY, the technology will displace more health than it delivers. There is also nothing in NICE policy to prevent managed access arrangements from being agreed at costs that far exceed this basic threshold, and such drugs will often be evaluated according to prioritisation standards that further inflate what is considered an acceptable cost per QALY. Furthermore, experience has shown that rescinding access to a technology that patients have come to rely on is fraught with ethical and political challenges [86]. In practice, therefore, this standard is unlikely to further the goal of promoting population health, though it is likely to be very successful in supporting innovation.

If we accept that NICE’s concern for innovation is motivated not just by a desire to promote population health, but also by a need to support the commercial life sciences, then several other aspects of NICE policy begin to cohere around this value. The asymmetric prioritisation that poses such a challenge to fairness can be easily justified according to this conception of innovation, because while patented new technologies are of significant commercial value to the life sciences industry, the existing NHS interventions displaced to fund them are likely to be older and of less economic importance. Such a policy does not cohere with the goal of promoting population health or with NICE’s substantive views of fairness, but it does cohere with its goal of supporting the life sciences. A similar argument might be used to justify NICE’s various prioritisation standards, which do not cohere well with one another or with other elements of the primary cluster, but which tend to facilitate the routine adoption of commercially important cancer drugs and other expensive technologies that generate profit and growth for the life sciences. A concern for supporting a thriving life sciences industry also provides plausible justification for NICE’s continued use of a cost-effectiveness threshold which, according to the best available evidence, is highly likely to underestimate the opportunity cost of NICE’s recommendations.

This is not to suggest that no positive relations exist between the goals of supporting innovation and promoting population health. The technological and commercial success of the life sciences sector has contributed vastly to population health over the last 200 years, so these goals might to some extent be considered mutually supportive, particularly when taking a long-term perspective. But drug companies are, quite legitimately, motivated by goals aside from the promotion of population health – most obviously courtesy of their legal and professional duty to maximise shareholder returns – and the government’s aims in supporting this sector are equally motivated by economic and political as well as health considerations. Indeed, if these goals were synonymous then NICE’s commitment to supporting innovation would be redundant; the life sciences industry would be the natural beneficiary of any policies aimed at promoting population health. As it stands, however, this is not the case and elements of NICE policy aimed at promoting innovation do not, as currently specified, fully cohere with the goal of promoting population health. Depending on how these competing priorities are balanced and acted on by appraisal committees, this introduces the potential for substantial dissonance between the primary and secondary cluster and gives reason to question the extent to which NICE’s approach is morally justified.

### Liberty

One final substantive value that forms part of NICE’s approach is that of liberty, or freedom.

In *SVJ*, NICE formally subscribes to the principle of autonomy and notes that “patient choice” is an “important” consideration for the NHS and its users [9]. However, it insists that such notions do not take precedence over matters of fairness and “should not mean that NHS users as a whole are disadvantaged by guidance recommending interventions that are not clinically and/or cost effective” (*ibid*.). In *Principles*, NICE similarly asserts that “not everything people might want will necessarily be available” [11]. However, it emphasises the need for committees to “balance” concerns about efficiency with “respect for individual choice”, implying some role for the latter in decision-making (*ibid.*).

In practice, it is unclear what this role might be. While much of NICE’s approach is centred on the goal of promoting population health for the good of society, patient choice rests on the very different goal of promoting liberty for the good of the individual. When these goals come into conflict, as they often will, it is not clear how they might be coherently balanced. If a committee is faced with a technology that offers no population-level benefit over existing treatment and comes at greater cost, then concern for population health implies that it should be rejected. Concern for individual choice implies that it should be recommended. A practical compromise might be to assign some value to choice itself, allowing such technologies to be recommended as long as the opportunity cost is not too great – indeed, NICE’s use of managed access arrangements might be understood in part as an attempt to achieve such a compromise. But, as has already been discussed, this approach potentially disadvantages other NHS users in a way that appears neither fair nor reasonable, as these values are currently specified, and is illogical insomuch as it deprives other NHS users of liberty by preventing them from accessing interventions from which they would benefit.

A more straightforward alignment exists between NICE’s concern for individual choice and its general support for innovation, both of which share (neo)liberal roots and to some extent decouple a technology’s value from its ability to improve health. Indeed, consumer choice in the healthcare arena can be seen as an important driver of pharmaceutical innovation and potentially provides some justification for NICE’s apparent preference for adopting new, branded health technologies over retaining older, more well-established interventions. A positive relation might therefore be drawn between the value of liberty and the secondary cluster, which similarly views a situation in which patients are able to choose between a variety of (commercially productive) interventions as preferable to one in which access is limited to those that make the greatest contribution to population health.

## Discussion

This analysis demonstrates that much of NICE technology appraisal policy coalesces around a set of mutually supportive substantive values: fairness, reasonableness, lawfulness, legitimacy and health itself. At an abstract level, these values are highly coherent and interact positively to support NICE in its goal of promoting population health. However, NICE policy also contains several sources of dissonance which undermine coherence and prevent equilibrium from being fully established across its normative commitments.

Four main aspects of NICE policy pose a challenge to coherence. First is the principle of cost-effectiveness and NICE’s specification of this principle through a threshold that, according to the best available evidence, substantially underestimates NHS opportunity cost, giving rise to outcomes contrary to several aspects of NICE’s conception of fairness. Second is NICE’s collection of overlapping prioritisation standards, which do not obviously align with any consistent principle, and which again give rise to outcomes that appear contrary to fairness. Third is NICE’s inconsistent conception of what constitutes a reasonable decision in the absence of strong evidence and the dissonance that arises from the attempt to commit simultaneously to conservative and pro-innovation strategies for managing uncertainty. And fourth is NICE’s concern for innovation, which appears to be motivated in part by political and economic priorities that do not always relate positively with the primary cluster’s main goal of promoting population health.

These sources of persistent dissonance across NICE policy raise questions about the extent to which NICE’s approach is morally justified. But if this is the case, then a key question remains: why do NICE’s decisions continue to be accepted as legitimate?

### Why are NICE’s decisions accepted as legitimate?

There are several potential answers to this question.

The first and most obvious is that the public is simply unaware that these sources of dissonance exist and that NICE’s approach may therefore be morally problematic. Ours is the first attempt to systematically subject NICE policy to empirically based moral evaluation and outside of specialist academic, policy and patient communities, knowledge and understanding of NICE’s detailed approach is likely to be low. Evidence suggests that media reporting of NICE decision-making tends to be uncritical, with the most common narrative centring on the need for greater NHS access to potentially beneficial technologies [87–90]. NICE also takes care to describe its approach in a way that appears coherent. *Principles*, which has been NICE’s main articulation of “the morals, ethics and values” underpinning its work since 2020, has been shown to offer an incomplete account of NICE’s approach and avoids acknowledging some of the more contentious standards embedded within it [44]. But casual readers of this and other publicly oriented statements are likely to take them at face value and will almost certainly not spend time studying the technical documents in which the sources of dissonance highlighted by this study are embedded.

A second reason for society’s seemingly passive acceptance of NICE’s decisions is that they are typically positive. According to recent statistics, since 2000 84% of NICE appraisals have led to the technology in question being either fully recommended, partially recommended or recommended for use via the Cancer Drugs Fund [91]. The main beneficiaries of these decisions are drug manufacturers and those patients who benefit from access to such technologies, whose interests are often represented by organised advocacy groups and/or charities. These stakeholders are often influential actors in UK health policy and have shown themselves willing to mobilise their resources and social and political capital in challenging negative decisions [92–95]. However, they are unlikely to challenge positive decisions on the grounds that NICE’s approach is not morally justified. In contrast, those who experience the opportunity cost associated with such decisions are unidentified patients who are highly dispersed and likely unaware of the impact that NICE’s decisions have on their interests. With the exception of the NHS itself, therefore, which has historically only challenged NICE’s decisions when these have carried significant financial and operational burdens [96], and Parliament, which has periodically expressed concern about NICE’s approach but has not gone so far as to mandate change [97–99], there is no organised group motivated to hold NICE to account in ensuring that its actions are coherent. The current approach – in which “we approve the majority of medicines and treatments” [91] – might therefore be seen as a pragmatic (if morally suspect) compromise which allows outspoken and influential interests to be appeased at a cost low enough (or imperceptible enough) to be accepted by the rest of society.

Positive decisions are also highly unlikely to be formally challenged through appeal. In theory, any decision can be appealed if it meets NICE’s grounds for doing so. But in practice, practically all NICE appeals relate to negative or optimised recommendations and are brought by those groups who are disadvantaged by such decisions [100]. The grounds for appeal also do not allow NICE’s decisions to be overturned for substantive reasons, except where a decision can be shown to be either unlawful or ‘Wednesbury unreasonable’: that is, where the conclusions reached by an appraisal committee are “obviously and unarguably wrong, illogical, or so absurd that a reasonable advisory committee could not have reached such conclusions” [55]^22^ This does not provide any route to challenge decisions on the grounds that they are substantively unfair, for example, or are based on insufficient evidence, and it also does not allow for particular aspects of NICE’s approach to be challenged on the grounds that they are incoherent or otherwise unjustified. Although, as a public body, the lawfulness of NICE’s actions can be adjudicated through judicial review, the courts have also shown themselves unwilling to challenge the substantive nature of NICE’s decisions [101, 102]. There is therefore, in practice, very limited opportunity for substantive moral aspects of NICE’s approach – either policy or practice – to be formally challenged.

A further possible reason for the public’s continuing acceptance of NICE’s decisions is the high level of legitimacy bestowed on it internationally [103] and through other aspects of its approach. As this analysis has shown, the primary cluster is highly coherent and rests on values that are both morally plausible and, for the most part, likely to be well supported by the public. NICE’s approach also rests on a strong conception of procedural fairness and has many procedural strengths – in particular, the independence of its appraisal committees and the relative transparency of its decision-making. Such strengths, and NICE’s well-established reputation as a competent priority-setter, act as a significant defence against claims that NICE’s approach lacks moral legitimacy. However, this defence is arguably weakening: increasing levels of data redaction in NICE appraisal documentation [104] and changes to NICE’s funding structure, (the Institute now receives much of its income from industry [105]), arguably pose significant threats to NICE’s transparency and independence, and NICE’s reputation – though good – is not immune to damage through morally questionable decision-making. NICE’s continued legitimacy in the future may therefore not be assured.

Finally, it is possible that, in their judgements made in response to individual cases, appraisal committees can exercise sufficient discretion to coherently balance elements of NICE’s approach that – on paper – are problematic. This may help to maintain equilibrium and prevent questionable aspects of NICE’s moral system from being exposed to public scrutiny. For example, committees might act to ‘smooth’ the disparity in policy between NICE’s substantial prioritisation of very rare conditions and non-prioritisation of similar but (merely) rare conditions through a willingness to somewhat exceed the £20,000-£30,000/QALY threshold in the latter case. However, NICE’s recent move towards a more algorithmic approach to decision-making might limit committees’ discretion to balance competing commitments in this way [43]. Further empirical work is needed to explore how committees interpret and act upon NICE policy and the extent to which NICE policy and practice, taken together, can be considered to exist in a state of equilibrium.

### How might NICE’s approach be made more coherent?

Given the moral complexity of the priority-setting endeavour, the political pressure under which NICE operates and the extent to which its future is dependent on the continued support of influential stakeholders from across government, industry and society, it may not be feasible for NICE to make the changes required to bring its policy entirely into equilibrium. But equilibrium remains a worthwhile goal and this analysis highlights several ways in which potential sources of dissonance within NICE policy might be addressed with a view to improving coherence. For example:

- By reflecting on what is meant by the concept of ‘need’ and by formulating a morally plausible principle of prioritisation that could be used to coherently reconcile prioritarian aspects of NICE’s approach;
- By developing a consistent view of what reasonableness requires in terms of the use of evidence, and by ensuring that related standards – particularly those concerning managed access – are specified in ways that support the values of fairness and reasonableness;
- By further contemplating what types of considerations are relevant to healthcare priority-setting, and by ensuring that standards specifying the role these should play in different contexts are morally plausible and coherent;
- By formulating a definition of innovation that reflects NICE’s goals and the normative value associated with innovative technologies, and by reflecting on how a commitment to innovation could be specified in ways that remain in equilibrium with the values of fairness, reasonableness, lawfulness, legitimacy and health;
- By reviewing how NICE’s normative approach is publicly articulated and by ensuring that this articulation fully meets the demands of procedural fairness.

Further reflection on these matters – and a willingness by NICE to adjust policy to improve its coherence – could do much to address the potential for dissonance in NICE’s approach and would give NICE stronger grounds for claiming that its actions are morally justified. However, as has already been acknowledged, NRE holds limited justificatory power compared with a wider equilibrium that incorporates relevant background theories and reflects societal values more broadly. The coherence of NICE’s approach could therefore be further strengthened by engaging the public in this process and by seeking to move towards WRE through meaningful public debate on each of these issues. In seeking to ensure coherence, it is also important that NICE’s decision-making committees, both internal and external, are peopled by individuals representing a diversity of views, who possess skills in normative reasoning and display the characteristics of what Rawls termed a ‘competent moral judge’: intelligence, empathy and impartiality [106].

Given that NICE apparently continues to draw political and public support in its role as national healthcare priority-setter, it could be argued that no change is necessary and there is no need to depart from its current approach. But a foundational assumption of applied ethics is that society, and its institutions, should seek to act in a way that is morally legitimate. Given NICE’s stated desire to “act ethically and with the highest standards of integrity, quality, probity, openness and accountability” [107] it is therefore appropriate to highlight changes that could improve the coherence of its approach and might provide it with stronger grounds for claiming that its actions are morally justified.

### Study limitations and future work

Our study has some limitations. First, the findings relate to policy as of 31 December 2021 and do not take account of subsequent changes to NICE’s approach, including those detailed in its new *Health Technology Evaluation Manual*, published in January 2022. A recent review of this manual concluded that “﻿most of the updates correspond to clarifications, formalization of best practice, and guidance for new challenges in line with existing principles” and thus constitute a “relatively modest” update to NICE’s procedures [41]. In at least one instance, the updates appear to amplify existing sources of dissonance: the manual will allow for greater tolerance of uncertainty when evaluating innovative technologies (a designation that remains undefined), increasing the potential for innovation to be supported in ways detrimental to overall population health (*ibid.*). However, further work would be required to better understand the implications of these changes for the coherence of NICE’s approach.

Second, alternative mappings of the identified normative commitments and the relations between them are possible. While our personal views and assumptions may have influenced the findings, we adopted several strategies to mitigate their impact, such as extensive double-coding and peer debriefing with experts not on the authorship team. More importantly, reflective equilibrium is a process and the conclusions we draw here should be viewed as a “resting point”, not an end point [30, 108]; for example, future engagement with decision-makers at NICE may uncover implicit principles, informal standards not codified in policy [49] or alternative explanatory relationships between commitments that can help resolve some of the instances of dissonance discussed here.

Finally, as highlighted earlier in the paper, the scope of this study is limited to policy and does not extend to a consideration of the case-based judgements made by NICE’s appraisal committees in practice, which are important to the overall coherence of NICE’s approach. This somewhat reduces the reliability of the conclusions that can be drawn from this analysis, given that committees may hypothetically act in ways that resolve the identified sources of dissonance. However, in considering the many values, principles and standards embedded in policy, we are nevertheless able to draw conclusions about the extent to which policy itself is coherent, which, we argue, carries some justificatory power, particularly given that a clear articulation of its reasons for decision-making forms one of NICE’s core normative principles. NICE appraisal committees have some discretion to exercise judgement in their application of NICE policy, but this discretion is not unlimited and is substantially constrained by the algorithmic nature of certain aspects of NICE’s approach [43]. As such, it is highly likely that dissonance in NICE policy will lead to some dissonance in NICE practice and, even where committees successfully balance normative commitments in a way that maintains equilibrium, the ambiguity that such dissonance generates is likely to erode transparency, which is itself to the detriment of coherence. Further research to explore NICE’s case-based judgements – and to establish the extent to which policy and practice are in equilibrium – is currently underway and will be published in due course. However, findings that have emerged to date do not suggest that the sources of dissonance identified in this study of policy are fully resolved in practice.

## Conclusion

There is much to celebrate about NICE. For many years it has performed a difficult role, maintaining its reputation as a legitimate priority-setter despite the unpopular decisions that is has sometimes had to make. This longevity might in part be attributed to NICE’s strong moral foundations. The values at the heart of its approach are highly coherent and provide a sound basis from which to strive towards the goal of promoting population health. But NICE’s approach has necessarily had to evolve in response to political, social and technological changes, and it appears to have done so in ways that have undermined coherence and given cause to question the extent to which it can be morally justified.

It is highly likely that NICE is aware of this issue. However, it has so far been able to protect itself from public challenge – and its decisions from rigorous ethical scrutiny – by choosing not to publicise the moral inconsistencies embedded within its approach and by acting in ways that primarily benefit those with the loudest voices. The interests of the UK Government, the life sciences industry and those patient advocacy groups most closely engaged in NICE’s work are aligned in their support for adopting new technologies, providing momentum for an approach increasingly centred on the goal of supporting innovation, even when doing so does not benefit population health overall.

The legitimacy of this approach may yet come under challenge, particularly if current NHS pressures continue to build. But even in the absence of such a prompt, NICE has made a commitment to act in a way that can be morally defended. This paper highlights areas for further attention and suggests ways in which NICE might seek to better honour that admirable commitment.

### Supplementary Information

**Additional file 1. **Documents included in analysis

**Additional file 2. **Code system and coding frequency (substantive only)

## Data Availability

The dataset supporting the conclusions of this article is included within the article. A copy of the analytical guide is available on request.

## Footnotes

1 See, for example, the extended debate in the Journal of Medical Ethics between philosopher John Harris and authors associated with NICE about the appropriateness of NICE’s substantive principles, between 2005 and 2007 [19–27].

2 The term ‘reflective equilibrium’ refers to both the method endorsed by Rawls and the end state to which this method is directed towards.

3 This does however remain an open question. We are not aware of any work that has specifically explored this issue. However, we see moral justification as a matter of degree; as such, our primary interest is the extent to which NICE’s approach is coherent, rather than the binary question of whether it meets some defined standard of NRE. That said, it is our view that – given sufficient motivation and opportunity for reflection – it should be possible for an institutional priority-setter such as NICE to develop an approach that rests on mutually supportive normative commitments and is free from substantial persistent dissonance, thus meeting most interpretations of NRE. We base this view on several factors. First, although it is generally accepted that WRE is a largely aspirational goal, the more limited achievement of NRE is usually held to be attainable – this is one reason why the process of RE is regularly used as a tool in practical ethics. Second, the view that NRE is achievable in this context is empirically supported because equilibrium has been demonstrably attained in a wide range of scenarios, many of which are of comparable normative complexity to that of healthcare priority-setting (for example, in studies considering ethical issues in critical care nursing, research on human embryonic stem cells and environmental impact assessment [36]). Third, it seems reasonable to suggest that NICE’s approach is capable of being brought into reflective equilibrium because our analysis comes relatively close to doing so – at least across the values, principles and standards formally embedded in policy [35, 36].

4 It may be that apparent sources of dissonance within a narrow reflective equilibrium might be resolved if wider considerations were taken into account. For example, it might be argued that apparent inconsistencies that arise from a desire to satisfy the needs of different patient groups is justified with reference to the value of democracy and the principle of democratic representation. However, our claim here is not that it is impossible to establish equilibrium across NICE’s approach, taking such wider considerations into account; it is merely that there currently exist instances of dissonance that prevent us from fully establishing NRE (as we conceive of it) across NICE policy. Moreover, if NICE does rely on unacknowledged values (such as democracy) in justifying some of its more specified principles and standards, then its commitment to transparency (under the AfR framework) would require it to acknowledge this fact. On these grounds at least, coherence with its commitment to transparency would be undermined by reliance on such wider, unstated commitments.

5 The study does not consider more recent changes to NICE policy including those implemented in January 2022. However, a critical analysis of these changes concludes that they are “modest in impact” and are therefore unlikely to challenge the main findings of this study [41].

6 Case-based judgements do not feature in this analysis because, by definition, they relate to individual cases and therefore form part of practice, not policy.

7 Van Thiel and Van Delden use the following example, taken from Bonjour, to demonstrate what coherence requires: *Proposition A: this chair is brown; electrons are negatively charged; today is Thursday.* *Proposition B: all ravens are black; this bird is a raven; this bird is black.* Neither of these two propositions are logically inconsistent. But in proposition A consistency results from the fact that its components are almost entirely unrelated to each other – although not inconsistent, they contain no relations of mutual support. This is not the case in proposition B, in which there are strong positive inferential relations between the premises. In our analysis, we would not consider the components of proposition A to be dissonant. However, the same would not be said for proposition C: *Proposition C: Brown chairs are better than black chairs; we should purchase some black chairs. * These premises are not logically inconsistent and there may be a valid reason for wanting to purchase black chairs. But this reason does not lie in the fact that brown chairs are better than black chairs. A negative inferential relation appears to exist between these two normative statements and, *prima facie,* they would therefore be classified in our analysis as dissonant  [40, 51].

8 It is an open question whether fair-mindedness requires an individual’s moral beliefs to also be coherent. We do not assume that this is the case.

9 This differs from a fully maximising approach in that it considers technologies individually and does not entail reviewing the entire NHS offering to decide what allocation would be most efficient. The latter approach was taken in the famous Oregon plan which involved a comprehensive ranking of health interventions based on relative cost-effectiveness [57].

10 This practice itself reflects several normative and empirical principles, the details of which need not concern us here. See the HM Treasury’s ‘Green Book’, Chapter 14 for a full exposition [63].

11 In the case to which it was first applied, this reduction in discount rate had the effect of improving the technology’s cost-effectiveness from £56,700/QALY to £36,000/QALY, allowing an initial rejection to be reversed and the drug to be recommended as a “cost-effective use of NHS resources”  [64].

12 It may be that committees act to ‘smooth’ some of these cut-offs by applying prioritisation criteria somewhat flexibly. Such questions are being addressed in the second stage of this work. However, even if committees do apply some flexibility, the algorithmic nature of these standards will still lead to fairly binary outcomes, with some groups receiving significant priority and others not.

13 The relevant principle, as stated in *SVJ*, is open to interpretation, so it is not possible to say with certainty whether these standards might be considered to cohere with it. As argued elsewhere in this paper, such ambiguity is itself at odds with NICE’s commitment to AfR which requires transparency.

14 The argument that the HST programme effectively applies the ‘rule of rescue’ is made in full elsewhere [68].

15 *Principles* states that, while superseded, *SVJ *“remains relevant to our work”.

16 This argument has also been made elsewhere [44].

17 This may include those patients who will have access to the new technology, but only if it is later shown to be more clinically effective than the alternatives.

18 Though committees have discretion to take this into account in individual non-HST cases, there is no requirement to do so. Even if a committee did deem this to be a relevant consideration in a specific case, it is unlikely that such discretion would extend to exceeding the usual threshold by up to a magnitude of three, as in the case of the HST programme.

19 NICE argues that it “has established rigorous systems to protect against ageist decisions, has no track record of ageism and is well placed to meet the requirements of new UK equality legislation” in relation to the non-discrimination of older patients. However, it acknowledges that the preferential treatment of technologies indicated for children is also a potential form of age discrimination and is one that “NICE will continue to consider” going forward [84].

20 Empirical evidence shows that NICE appraisal committees do, indeed, recommend technologies that exceed the usual cost-effectiveness threshold on the grounds of innovation, even when no uncaptured health benefits have been identified [45].

21 This assumes that the £20,000-£30,000/QALY threshold is an appropriate specification of the principle of cost-effectiveness, which, as previously discussed, is not supported by the available evidence.

22 This term refers to *Associated Provincial Picture Houses Ltd v Wednesbury Corporation (1948) 1 KB 223* and is a standard used in assessing an application for judicial review of a public authority's decision. It is a stricter test than merely showing that a decision was unreasonable.
