# Supplementary material for: An empirical ethics study of the coherence of NICE technology appraisal policy and its implications for moral justification
Source: BMC Med Ethics. 2024 Mar 6;25:28. doi: 10.1186/s12910-024-01016-0 (PMC10918908; doi:10.1186/s12910-024-01016-0)
Supplement: Supplementary file 1 — Additional file 1. Documents included in analysis [file 12910_2024_1016_MOESM1_ESM.docx]

Appendix 1: Documents included in analysis

| **No.** | **Document title** | **Year** | **Document type** |
| --- | --- | --- | --- |
| 01. | Guide to the methods of technology appraisal | 2013 | Technical guide |
| 02. | Highly Specialised Technologies programme: Interim process and methods | 2013 | Technical guide |
| 03. | Guide to the technology appraisal and highly specialised technologies appeal process | 2014 | Technical guide |
| 04. | Guide to the processes of technology appraisal | 2014 | Technical guide |
| 05. | Rapid re-consideration of drugs currently funded through the Cancer Drugs Fund | 2016 | Technical guide |
| 06. | Technology Appraisal and Highly Specialised Technologies Programmes: Procedure for varying the funding requirement to take account of net budget impact | 2016 | Technical guide |
| 07. | Interim Process and Methods of the Highly Specialised Technologies Programme: Updated to reflect 2017 changes | 2017 | Technical guide |
| 08. | PMG19 Addendum A: Final amendments to the NICE technology appraisal processes and methods guides to support the proposed new Cancer Drugs Fund arrangements | 2016 | Addendum to technical guide |
| 09. | Cost comparison: Addendum to the Guide to the methods of technology appraisal) | 2016 | Addendum to technical guide |
| 10. | Guide to the processes of technology appraisal April 2018: Interim addendum – Procedures for the review of commercial and managed access requests | 2021 | Addendum to technical guide |
| 11. | Fast track appraisal: addendum to the guide to the processes of technology appraisal | No date | Addendum to technical guide |
| 12. | Social value judgements: Principles for the development of NICE guidance (2nd edition) | 2008 | Ethical guide |
| 13. | Our Principles | 2020 | Ethical guide |
| 14. | Pharmaceutical Price Regulation Scheme 2014 – implications for NICE | 2015 | Position statement |
| 15. | NICE’s biosimilars position statement | 2016 | Position statement |
| 16. | NICE and the life sciences industries | 2017 | Position statement |
| 17. | NICE position statement: Chemotherapy dose standardisation | 2018 | Position statement |
| 18. | Position statement on use of the EQ-5D-5L value set for England | 2019 | Position statement |
| 19. | NICE’s biosimilars position statement | No date | Position statement |
| 20. | NICE Charter | 2021 | Other |
