# Supplementary material for: An empirical ethics study of the coherence of NICE technology appraisal policy and its implications for moral justification
Source: BMC Med Ethics. 2024 Mar 6;25:28. doi: 10.1186/s12910-024-01016-0 (PMC10918908; doi:10.1186/s12910-024-01016-0)
Supplement: Supplementary file 2 — Additional file 2. Code system and coding frequency (substantive only) [file 12910_2024_1016_MOESM2_ESM.docx]

Appendix 2: Code system and coding frequency (substantive only)

| **SUBSTANTIVE CODE** | **CODING FREQUENCY** |
| --- | --- |
| **V1. LEGITIMACY** | 9 |
| P1. Decisions should be legit because NHS is publicly funded | 2 |
| P2. Decisions should be legit because they have significant impacts | 2 |
| P3. Legitimacy requires that NICE be accountable for its decisions | 7 |
| P4 Legitimacy requires opportunities for participation | 11 |
| P5. Legitimacy requires adoption of a pluralistic approach | 3 |
| P6. Legitimacy requires that public views be taken into account | 2 |
| S1. Normative principles informed by Citizens Council | 1 |
| S2. Topic selection: topics should be of importance to stakeholders | 4 |
| **V2. HEALTH** | 0 |
| P7. Primary responsibility is to deliver health via NHS resources | 3 |
| P8. Decisions should promote the health of the population as a whole | 8 |
| P9. Decisions should take account of health benefits and harms | 4 |
| P10. Decisions should support the provision of high-quality care | 9 |
| S3. Topic selection criterion: Answers relevant clinical questions | 1 |
| S4. Topic selection criterion: No existing NICE guidance | 1 |
| S5. Topic selection criterion: Not accepted based on other guidance | 1 |
| S6. Topic selection criterion: Not covered by other bodies | 1 |
| S7. Topic selection consideration: impact on population health | 3 |
| S8. Topic selection consideration: Need for consensus view | 3 |
| S9. Topic selection consideration: Inappropriate variation in use | 2 |
| S10. HST criteria: Significant need for national commissioning | 1 |
| P11. Health is a measure of both quality and quantity of life | 5 |
| **V3. FAIRNESS** | 12 |
| P12. Utilitarian position: fairness as efficiency | 7 |
| P13. Decisions should seek to maximise health | 6 |
| P14. Decisions should show regard for balance of costs & benefits | 17 |
| P15. Decisions should be informed by cost-effectiveness | 31 |
| S11. Technologies are cost-effective if benefits > opportunity costs | 8 |
| S12. £20-£30k threshold reasonable reflection of opportunity cost | 1 |
| S13. Not appropriate to adjust threshold based on PPRS | 2 |
| S14. Cost-effectiveness calculated via standard reference case | 5 |
| S15. Preference for cost-utility analysis | 11 |
| S16. No precise cost-effectiveness threshold | 2 |
| S17. <£20,000/QALY, benefits generally sufficient to justify OC | 6 |
| S18. £20-30,000/QALY, specific reasons needed | 5 |
| S19. >£30,000/QALY, benefits generally not sufficient to justify OC | 3 |
| S20. All QALYs valued equally in reference case | 4 |
| S21. Guided by comparison with established NHS practice | 5 |
| S22. Perspective on benefits: all direct health effects | 1 |
| S23. Utility measures based on public preference | 2 |
| S24. Measurement of HRQoL reported directly by patients | 2 |
| S25. EQ-5D(-3L) preferred tool for valuing health effects in adults | 7 |
| S26. Use of the EQ-5D-5L is not recommended. | 2 |
| S27. Alternatives to EQ-5D used by exception | 4 |
| S28. Estimates of treatment effect based on systematic review | 1 |
| S29. Statistical methods used to adjust for trial cross-over | 2 |
| S30. Clinical end points preferred to surrogate end points | 1 |
| S31. Consideration of non-health outcomes for individuals | 4 |
| S32. Consideration of wider benefits to public bodies | 5 |
| S33. Perspective on costs: NHS/PSS | 4 |
| S34. Costs valued using relevant prices | 6 |
| S35. PPRS rebates not taken into account under relevant price | 3 |
| S36. Blended prices only used by exception | 1 |
| S37. Consideration of price reductions | 2 |
| S38. Costs to other services explored by exception | 1 |
| S39. Exclusion of value added tax (VAT) | 1 |
| S40. Exclusion of unrelated future costs | 1 |
| S41. Exclusion of productivity costs | 2 |
| S42. Exclusion of PPRS repayments | 1 |
| S43. Consideration of care by family members etc. | 2 |
| S44. Consideration of costs of companion diagnostics | 2 |
| S45. Consideration of costs borne by patients | 1 |
| S46. Consideration of related future costs | 1 |
| S47. Consideration of infrastructure costs | 1 |
| S48. Time horizon sufficient to reflect relevant outcomes | 4 |
| S49. Annual discount rate of 3.5% for costs and benefits | 2 |
| S50. Consideration of subgroup analyses | 8 |
| S51. Subgroup recommendations only when based on clear evidence | 1 |
| S52. Subgroups must be justified | 5 |
| S53. Cost-based subgroups generally considered acceptable | 1 |
| S54. Cost-based subgroups excluded if based on geography | 1 |
| S55. Cost-based subgroups excluded if based on social characteristic | 1 |
| S56. Subgroups based on response justified in some circumstances | 3 |
| S57. Consideration given to robustness, plausibility, feasibility | 3 |
| S58. Consideration given to cost-effectiveness | 3 |
| S59. Consideration given to fairness re: withdrawing treatment | 3 |
| S60. Consideration of uncaptured health gain | 3 |
| S61. Value for money defined more broadly for HSTs | 2 |
| P16. Technologies should not be recommended if not cost-effective | 6 |
| S62. Rejection of the rule of rescue | 3 |
| P17. Decisions should be based primarily on cost-effectiveness | 1 |
| P18. Decisions should consider factors aside from cost-effectiveness | 9 |
| S63. Consideration given to non-health objectives of the NHS | 3 |
| P19. Technologies should not be recommended if costs are unreasonable | 2 |
| P20. Prioritarian position: fairness as priority to the worst-off | 2 |
| P21. Decisions should prioritise those with greatest clinical need | 4 |
| P22. Prioritise the needs of those suffering from severe diseases | 1 |
| S64. Consideration given to degree of morbidity/clinical disability | 2 |
| S65. HST criteria: Condition is chronic and severely disabling | 1 |
| P23. Prioritise the needs of those with cancer | 1 |
| S66. Preferential treatment of cancer drugs through the CDF | 1 |
| S67. Plausible potential for satisfying the criteria for routine use | 3 |
| S68. Uncertainty can be addressed through data collection/reappraisal | 1 |
| P24. Prioritise the needs of those nearing the end of life | 0 |
| S69. QALYs valued more highly at the end-of-life | 4 |
| S70. End-of-life QALY weighting: short life expectancy | 2 |
| S71. End-of-life QALY weighting: life extension | 2 |
| S72. End-of-life QALY weighting: small patient population | 1 |
| S73. End-of-life QALY weighting: assumption of full quality of life | 2 |
| S74. End-of-life QALY weighting: maximum weight of 1.7 | 2 |
| S75. End-of-life QALY weighting: based on robust evidence | 2 |
| S76. End-of-life QALY weighting: inferred from surrogate outcomes | 2 |
| S77. End-of-life QALY weighting: not necessarily used as comparators | 2 |
| P25. Prioritise the needs of those with very rare conditions | 3 |
| S78. Higher threshold used in evaluating HSTs | 1 |
| S79. < £100,000/QALY, benefits generally justify costs | 3 |
| S80. HST criteria: Target patient group is clinically distinct | 1 |
| S81. HST criteria: Technology has potential for life-long use | 1 |
| S82. HST criteria: Technology has very high acquisition cost | 1 |
| S83. HST criteria: Target patient group is small and specialised | 1 |
| P26. Prioritise technologies that offer large health benefits | 2 |
| S84. Special discount rate applied in exceptional circumstances | 2 |
| S85. Special discount rate: high severity of illness | 2 |
| S86. Special discount rate: health significantly restored | 3 |
| S87. Special discount rate: benefits sustained over very long period | 2 |
| S88. Special discount rate: high likelihood of long-term benefits | 2 |
| S89. Special discount rate: no significant irrecoverable costs | 2 |
| S90. Magnitude of benefit QALY weighting for HSTs | 2 |
| S91. £100,000-300,000/QALY based QALY weighting and other factors | 3 |
| S92. Magnitude of benefit QALY weighting: compelling evidence | 1 |
| P27. Prioritise conditions with few alternative treatments | 0 |
| S93. Consideration of current treatment options | 4 |
| P28. Prioritise conditions suffering underinvestment | 1 |
| P29. Egalitarian position: fairness as equality | 5 |
| P30. Equality of treatment: act to treat all patients equally | 5 |
| S94. No special consideration for orphan drugs | 1 |
| S95. Non-consideration of individual behaviour and/or stigma | 4 |
| S96. Non-consideration of sexual orientation | 1 |
| S97. Non-consideration of sex/gender | 2 |
| S98. Non-consideration of age | 3 |
| S99. Non-consideration of race | 2 |
| S.100 Non-consideration of socioeconomic status | 2 |
| P31. Equality of opportunity: act improve equality of outcome | 11 |
| S101. Act to reduce socially based health inequalities | 8 |
| S102. Special consideration for the needs of the disabled | 1 |
| P32. Protect the interests of both current and future NHS patients | 2 |
| P33. Avoid unlawful and unfair discrimination | 5 |
| S103. Respect for parity of esteem (mental and physical health) | 2 |
| P34. Decisions should foster solidarity across the population | 3 |
| P35. Procedural position: fairness as fair process | 4 |
| S104. Grounds for appeal: NICE has failed to follow a fair process | 4 |
| **V4. REASONABLENESS** | 0 |
| P36. Decisions should be reasonable and robust | 11 |
| P37. Decisions should reflect sound interpretation of evidence | 15 |
| P38. Decisions should be informed by robust analysis | 1 |
| S105. Evaluation conducted according to an agreed scope | 1 |
| S106. Deviations from the scope for appraisal should be justified | 1 |
| S107. Evaluation conducted according to a standard reference case | 4 |
| S108. Deviations from the reference case should be justified | 3 |
| P39. Decisions should not be subject to bias | 5 |
| P40. Decisions should be informed by relevant knowledge & expertise | 11 |
| S109. Consideration given to patient perspective on outcomes | 5 |
| S110. Consideration given to clinical expert perspective on outcomes | 3 |
| P41. Decisions should be informed by adequate evidence | 12 |
| S111. Uncertainty taken into consideration in decision-making | 15 |
| S112. Higher tolerance of uncertainty for very rare diseases | 1 |
| S113. Caution exercised where uncertainty is great and ICERs high | 4 |
| S114. Greater weight given to high quality clinical evidence | 4 |
| S115. Greater weight given to high quality economic models | 2 |
| S116. Adequate evidence equates to robust evidence | 4 |
| S117. Benefits should be demonstrable | 9 |
| S118. Adequate evidence equates to best available evidence | 4 |
| S119. Benefits should be plausible | 6 |
| S120. Guidance reviewed in light of significant new evidence | 3 |
| S121. Technologies not recommended in the absence of adequate evidence | 8 |
| S122. Topics not considered in the absence of adequate evidence | 1 |
| P42. Recommendations for research used to address evidence gaps | 5 |
| S123. MAAs used to facilitate access while addressing uncertainty | 7 |
| S124. MAAs funded by the NHS | 1 |
| S125. Consideration given to costs and benefits of MAAs | 4 |
| S126. CDF used to facilitate access to uncertain cancer drugs | 2 |
| S127. MAAs used only when research is feasible and valuable | 7 |
| S128. 'Only in research' recommendations used to addess uncertainty | 4 |
| S129. Funding of OIRs not considered | 2 |
| S130. Consideration given to costs and benefits of OIRs | 1 |
| S131. OIRs consider likelihood of funding and completion | 2 |
| S132. OIRs consider value of evidence | 2 |
| S133. OIRs consider research timescale | 2 |
| P43. Level of scrutiny should be proportionate to level of risk | 2 |
| S134. Off label use considered only by exception | 6 |
| S135. Fast-track process adopted for low-risk technologies | 3 |
| S126. FT selection criteria: consequences of decision error | 3 |
| S137. FT selection criteria: ICER <£20k/QALY likely | 2 |
| S138. FT selection criteria: ICER >£30k/QALY unlikely | 2 |
| S139. FT selection criteria: robustness of evidence | 2 |
| S140. FT selection criteria: consistency with agreed scope | 2 |
| S141. FT selection criteria: precedent of approach | 1 |
| S142. FT selection criteria: patient population size | 1 |
| S143. FT selection criteria: budget impact | 1 |
| P44. Decisions should appropriately reflect relevant considerations | 3 |
| P45. Decisions should respect relevant precedent | 0 |
| S144. Decisions should be consistent with NICE policy | 15 |
| S145. Statement of compliance included in all guidance | 1 |
| S146. Decisions should be informed by similar cases | 7 |
| S147. Biosimilars treated as equivalent to originator product | 5 |
| S148. Biosimilars may be subject to independent evidence review | 1 |
| S149. CCA used where similar/> benefits anticipated at similar/< cost | 4 |
| S150. Decisions based on similarity of costs and benefits | 2 |
| S151. Relevant differences must be captured | 2 |
| S152. No systematic review of evidence on clinical effectiveness | 1 |
| S153. Data sources consistent with those previously used | 1 |
| S154. Relevance of some factors pre-defined via reference case | 1 |
| S155. Committee discretion to define/balance relevant considerations | 9 |
| P46. Decisions should not place excessive burden on NHS | 3 |
| S156. Lower tolerance of uncertainty where resource impact is high | 2 |
| S157. Budget impact considered in the appraisal of HSTs | 2 |
| S158. Budget impact considered in topic selection | 4 |
| S159. Recommendation not determined by budget impact | 1 |
| S160. Potential delay in funding where budget impact test is failed | 3 |
| S161. Delayed funding criterion: budget impact >£20m | 2 |
| S162. Delayed funding criteria: Option of last resort | 2 |
| S163. Delayed funding criteria: Plans in place for phased allocation | 4 |
| S164. Delayed funding criterion: account taken of severity / acuity | 2 |
| S165. Delayed funding criterion: consideration given to legislation | 2 |
| S166. Delayed funding criteria: delay proportional to budget impact | 4 |
| S167. Consideration of system impacts and limitations | 5 |
| P47. Decisions that are unreasonable should be open to challenge | 2 |
| S168. Grounds for appeal: Unreasonable in light of the evidence | 3 |
| **V5. LAWFULNESS** | 0 |
| P48. Decision-making should respect the rule of law | 32 |
| S169. Grounds for appeal: NICE has exceeded its powers | 3 |
| P49. Decision-making should comply with other formal commitments | 0 |
| P50. Decision-making should reflect NICE's formal 'Principles' | 1 |
| P51. Decision-making should respect the NHS Constitution | 4 |
| P52. Decision-making should align with the requirements of PPRS | 5 |
| P53. Decision-making should reflect instructions from Government | 2 |
| **V6. INNOVATION** | 3 |
| P54. Decisions should align with UK industrial policy | 8 |
| P55. NICE should maintain a positive relationship with industry | 6 |
| P56. Support global adoption of new health technologies | 3 |
| P57. Constructively manage tension between industry/NHS objectives | 1 |
| P58. Decisions should support innovation | 6 |
| P59. Act to facilitate access to the NHS market | 6 |
| S170. Special consideration for technologies of 'innovative nature' | 6 |
| S171. Use funding directive to drive uptake of products | 1 |
| S172. Collaborate with NHSE to manage financially challenging drugs | 1 |
| P60. Act to accelerate NHS access to effective technologies | 7 |
| S173. Provide routes to market for uncertain technologies | 2 |
| S174. Fast-track appraisal route open to low-risk technologies | 1 |
| P61. Seek to reduce risk for companies | 3 |
| S175. Support companies in making a reasonable return on investment | 3 |
| S176. Support companies in improving value proposition | 6 |
| **V.7 LIBERTY** | 6 |
| P62. Decisions should respect individual autonomy | 6 |
| P63. Respect for autonomy does not have absolute priority | 3 |
| **TOTAL: ALL CODES** | **963** |
